# Evaluation of MGIT 960 System for the Second-Line Drugs Susceptibility Testing of *Mycobacterium tuberculosis*

**DOI:** 10.1155/2013/108401

**Published:** 2013-03-28

**Authors:** Hyejin Kim, Minji Seo, Young Kil Park, Jae-Il Yoo, Yeong Seon Lee, Gyung Tae Chung, Sungweon Ryoo

**Affiliations:** ^1^Korean Institute of Tuberculosis, 168-5, Osong sangmyung 4 ro, Osong-eup, Cheongwon-gun, Chungbuk 363-954, Republic of Korea; ^2^Novartis Korea LTD, CD&MA, Namdaemunro 5-ga, Joon-gu 100-753, Republic of Korea; ^3^Centers for Infectious Diseases, National Institute of Health, Korea Centers for Disease Control and Prevention, 187 Osong sangmyung 2 ro, Osong-eup, Cheongwon-gun, Chungbuk 363-951, Republic of Korea

## Abstract

Many laboratories validate DST of the second-line drugs by BACTEC MGIT 960 system. The objective of this study is to evaluate the critical concentration and perform DST for the 2nd line drugs. We evaluated 193 clinical strains of *M. tuberculosis* isolated from patients in South Korea. Testing the critical concentration of six second-line drugs was performed by MGIT 960 and compared with L-J proportion method. The critical concentration was determined to establish the most one that gave the difference between drug resistance and susceptibility in MGIT960 system. Good agreement of the following concentrations was found: Concordance was 95% for 0.5 **μ**g/mL of moxifloxacin; 93.6%, 1.0 **μ**g/mL of levofloxacin; 97.5%, 2.5 **μ**g/mL of kanamycin; 90.6%, 2.5 **μ**g/mL of capreomycin; 86.2%, 5.0 **μ**g/mL of ethionamide; and 90.8%, 2.0 **μ**g/mL of *ρ*-aminosalicylic acid. The critical concentrations of the four drugs, moxifloxacin, levofloxacin, kanamycin, and capreomycin, were concordant and reliable for testing 2nd line drug resistance. Further study of ethionamide and **ρ**-aminosalicylic acid is required.

## 1. Introduction

Tuberculosis represents a major public health concern especially due to the increasing number of multidrug-resistant tuberculosis TB (MDR TB). Particularly in developing countries, extensively drug-resistant tuberculosis TB (XDR TB) continues to pose serious problem [1–5]. The increase in MDR/XDR TB rates prompts effective diagnostic methods so that appropriate treatments can be given to infected patients [6–8]. Many studies reported that MGIT 960 (Becton Dickinson Diagnostic System, Sparks, MD) provided reliable and rapid results in the detection and recovery of mycobacterium from clinical specimens and also the drug susceptibility testing (DST) of the TB isolates for the first line drugs isoniazid, rifampicin, ethambutol, streptomycin, and pyrazinamide [9, 10]. Recently, laboratories are facing great hindrances to provide DST for second-line drugs to ensure effective treatment of MDR/XDR TB by using MGIT 960 system [11–16]. However, in most of these studies, the tested numbers of second-line anti-TB drug were limited and the critical concentrations ranges of the second-line drugs were also discordant [11–13]. Despite the recommendation by WHO in 2008 for the use of liquid media for the second-line DST using MGIT 960, it is still unreliable due to the difficulty in determining the critical concentration [16]. It is not easy to calibrate newly developed DST methods using altered conditions. *In vitro* results of DST to second-line drugs were affected by criteria for measuring resistance such as the critical concentrations and critical proportions of drugs. For instance, DST with L-J solid media was established MICs that was defined as the drug concentration on which <20 colonies were found, while MIC in MGIT 960 was defined as the drug concentration at which the daily change in growth unit was less than that of the 1 : 100 control [17].

The Korean Institute of Tuberculosis (KIT) is a government operating research institute founded in 1970. KIT has been working as a main organization for conducting National TB program including diagnosis, basic research, and vaccine production. According to Korean Guidelines for Tuberculosis there are 5 practical tips for building treatment regimen for MDR-TB patients. Step 1 includes available 1st line drugs: pyrazinamide and ethambutol. Step 2: choose one of the injectable drugs: kanamycin, amikacin, streptomycin, capreomycin. Step 3: choose one of the quinolone drugs: levofloxacin, moxifloxacin, ofloxacin. Step 4: choose available drugs among group IV: *ρ*-aminosalicylic acid, cycloserine, prothionamide. Step 5: if 4 drugs are not composed until step 4 group V drugs can be considered: amoxicillin/clavulanate, clarithromycin, high-dose isoniazid, clofazimine, linezolid, thioacetazone (not for HIV patients).

The L-J proportion method has been used for both first- and second-line DST for a long time in Korea, but recently, a switch to MGIT 960 method has been initiated for first-line DST. In this study, we tested the various critical concentrations published second-line TB drugs, moxifloxacin, levofloxacin, kanamycin, capreomycin, ethionamide, and *ρ*-aminosalicylic acid and used L-J proportion method as a reference for comparison with the MGIT 960 system.

## 2. Materials and Methods

### 2.1. Strains

Cultures submitted to the Korean Institute of Tuberculosis from the Public Health Center in Korea were evaluated. A total of 193 clinical strains of *M. tuberculosis*, including 134 pansusceptible strains and 59 resistant strains whose drug susceptibility results were previously determined by L-J, were tested. All isolates were analyzed by each patient category with treatment history (Table 1). All isolates were freshly subcultured on L-J medium before being used and tested by the MGIT 960.

### 2.2. Chemicals

All drugs were in chemically pure powder form. Levofloxacin (LEV), Capreomycin (CPM), Kanamycin (KM), Ethionamide (ETH), and *ρ*-aminosalicylic acid (PAS) were obtained from Sigma-Aldrich (St. Louis, MO), and Moxifloxacin (MXF) was obtained from Bayer (Bayer Health Care AG, Germany). The powders were stored at −20°C in desiccators as recommended by the manufacturer. LEV, CPM, KM, and PAS were dissolved in deionized water (DW), MXF in 0.1 N NaOH with subsequent dilutions in DW, and ETH solubilized in DMSO with subsequent dilutions in DW. All stock solutions were sterilized by membrane filtration through 0.22 *μ*m-pore-size Millex-GS filter units (Millipore, Bedford, MA). All stock solutions were stored at −80°C in small aliquots. The frozen drug solutions were used immediately after thawing and the remaining was discarded and never stored in freezer again. Working solution was prepared freshly from the stock solution and the serial dilutions were carried out to achieve the desired concentrations.

### 2.3. Drug Susceptibility Test by the MGIT960 Method

MGIT DST was performed with strains grown from L-J slope, which were identified as *M. tuberculosis* with ZN staining. For the preparation of inoculums from L-J culture, 4 mL of Middlebrook 7H9 broth was added to a sterile tube with cap containing six to ten 2 mm glass beads. 1~2 colonies were extracted using a sterile loop from a growth culture that was not older than 14 days. The colonies were suspended in the Middlebrook 7H9 broth and vortexed for 2-3 minutes to break up the larger clumps. The suspension was left for 30 minutes to settle down the sediment to the bottom of the tube. Then, the supernatant fluid was transferred to another sterile tube and the suspension was left for another 15 min. Using 7H9 broth, the suspension was adjusted to a 0.5 McFarland standard. 1 mL of the adjusted suspension was diluted in 4 mL of sterile saline (1 : 5 dilutions). A 0.5 mL of the 1 : 5 dilutions was inoculated into tubes containing test drugs. In preparation of the second-line drug growth control tube, 0.1 mL of the previously mentioned suspension was pipetted into a total of 10 mL of sterile saline to prepare the 1 : 100 GC suspensions (1% growth control). The GC suspension was mixed thoroughly by gently inverting 3 to 4 times and then inoculated with 0.5 mL of the 1 : 100 GC suspensions into MGIT tubes. To interpret the results for 2nd drugs, the standard protocol recommended by the first-line drug manufacturers was followed for DST by the MGIT 960 method. When the growth unit (GU) of the growth control reaches 400 within 4–13 days, the GUs values of the drug-containing vials were evaluated. The GU of the drug-containing tubes were found 100 and >100; the result reported susceptible and resistant strains, respectively. Evaluated concentrations of each drug are listed in Table 2.

### 2.4. Drug Susceptibility Test by the L-J Proportion Method

L-J proportion method was the reference method for this study. All strains were previously tested on L-J medium by standard procedures at concentrations of MXF 2.0 *μ*g/mL, LEV 2.0 *μ*g/mL, KM 40 *μ*g/mL, CPM 40 *μ*g/mL, ETH 40 *μ*g/mL, and PAS 1.0 *μ*g/mL [18].

### 2.5. Genotypic Characterization

Strains that have shown discrepancy between the MGIT960 and L-J DST were retested in two methods. Also, we analyzed by DNA sequencing the key regions involved in the following genes; *gyrA* and *gyrB* for MXF and LEV, *rrs* for both KM and CPM, *tlyA* for CPM, and *inhA* promoter, *inhA*, *ethA*, and *ethR* for ETH. 

## 3. Results and Discussion

A total of 193 strains including 134 pansusceptible strains and 59 resistant strains with various drug susceptibility profiles (19 isolates were resistant to MXF and LEV, 16 isolates were resistant to KM, 11 isolates were resistant to CPM, 8 isolates were resistant to ETH, and 17 isolates were resistant to PAS according to the L-J method) were tested for susceptibilities for the second-line drugs. To determine the minimal inhibitory antimicrobial test concentrations (MICs) for 6 drugs, we analyzed twelve references having DST data using MGIT 960 for second-line drugs. To determine the critical concentration, it is based on the basis of distinguishable susceptible and resistant strains. The DST results for each 6 drugs are presented in Table 3. On testing both susceptible and resistant strains, the critical concentrations were obtained: 0.5 *μ*g/mL for MXF, 1.0 *μ*g/mL for LEV, 2.5 *μ*g/mL for KM, 2.5 *μ*g/mL for CPM, 5.0 *μ*g/mL for ETH, and 2.0 *μ*g/mL for PAS. Good agreement of critical concentration between the MGIT 960 and L-J proportion method results was observed for MXF, LEV, KM, and CPM. 

The MICs for MXF have been reported between 0.125 and 2.0 *μ*g/mL and resistant strains for this drug are characterized by a higher MIC [12, 13, 19, 20]. For example, MIC of clinical isolates and pansusceptible strains was reported on the basis of the MGIT960 method [12]. Also, it was reported that MICs of *gyrA* mutant strains were higher than those for pansusceptible and resistant strains [21]. We evaluated extended concentrations among 0.25~4.0 *μ*g/mL to determine MIC of resistant strain for MXF in this study (Table 2). 

For moxifloxacin, the agreement was 95% concordant at 0.5 *μ*g/mL. We found four strains with discordant result. One of those isolates was resistant at 1.0 *μ*g/mL as false resistant when repeated using the MGIT960 method. Three strains were susceptible when tested by L-J method, but resistant (at <0.5 *μ*g/mL) and susceptible (at ≥1.0 *μ*g/mL) when tested by MGIT960 (Table 4). Historically, those isolates were from each patient in the followup. To elucidate isolates with reduced susceptibility to MXF, we analyzed the mutation associated with resistance to MXF. Although either *gyrA* or *gyrB* mutations was not detected, those strains possibly carry a mutation which is located outside the QRDRs, or the resistance may be caused by other mechanisms. 

The MICs for LEV were recommended between 0.5 and 2.0 *μ*g/mL in previous papers [22, 23]. According to Sanders's paper, they tested MICs with both pansusceptible and resistant strain. MICs of resistant strains (2.0 *μ*g/mL) were higher than those for the pansusceptible strains (1.0 *μ*g/mL), which showed bimodal pattern like MXF [24]. We evaluated concentrations among 0.25~8.0 *μ*g/mL for LEV to determine MIC of resistant strains (Table 2). For levofloxacin, the agreement was 94% at 1.0 *μ*g/mL. Applying the 1.0 *μ*g/mL for the MGIT method, 18 of 19 resistant strains yielded resistant results and 37 of 40 susceptible strains yielded susceptible results (sensitivity 94.7%, specificity 92.5%). The three susceptible strains were susceptible at >1.0 *μ*g/mL when tested using the MGIT960 method. For the three susceptible strains, we analyzed gene sequence in those discordant strains and did not harbor the *gyrA* and *gyrB* mutations (Table 4). 

The MICs for KM were recommended 1.25~4.0 *μ*g/mL in the previous reports [19, 20]. Critical concentration of this drug is commonly recommended between 2.5 and 5.0 *μ*g/mL but it is greater than 20 *μ*g/mL in the case of using KM-resistant strains [6, 11, 19]. Range of MIC was chosen 0.625~10 *μ*g/mL to evaluate MICs of KM in our study (Table 2). 

For kanamycin, the use of the 2.5 *μ*g/mL critical concentration for the MGIT960 method led to yielding 97.5% concordance. 16 of 16 resistant strains yielded resistant results and 38 of 40 susceptible strains yielded susceptible results. The two discrepant strains did not harbor the *rrs* mutation (Table 4). We suggest the critical concentration for KM as a 2.5 *μ*g/mL to yield the best discrimination between susceptible and resistant strains. 

The MICs for CPM were reported 1.0~5.0 *μ*g/mL [19, 23, 25]. We evaluated MICs of CPM among 0.625~5.0 *μ*g/mL (Table 2). The concordance between both DST methods was 91% in capreomycin testing. Applying the 2.5 *μ*g/mL for the MGIT method, 10 of 11 resistant strains yielded resistant results and 37 of 40 susceptible strains yielded susceptible results (sensitivity 90.9%, specificity 90.3%). Three discordant susceptible strains were susceptible at >2.5 *μ*g/mL when tested using the MGIT960 method. Those strains were sequenced *rrs* and *tlyA* genes for genetic analysis but no mutations were detected in the two genes (Table 4). Resistant strain was found to be susceptible at >0.625 *μ*g/mL by MGIT 960. This isolate was from XDR-TB patient in the 4th follow-up sample. This strain also proved susceptible at >0.625 *μ*g/mL by MGIT 960 but resistant by L-J method. The resistance to fluoroquinolones was reported; aminoglycosides were developed during therapy [26]. Although it was isolated from patient during treatment, it seemed to obtain more susceptible results with the MGIT 960 instrumentation than with the comparative L-J method. 

ETH was recommended among 0.5~2.5 *μ*g/mL [19, 23, 25, 27]. We evaluated among 0.625~5.0 *μ*g/mL for ETH (Table 2). 

For ethionamide, we proposed 5.0 *μ*g/mL critical concentrations in the MGIT960. Using a concentration of 5.0 *μ*g/mL of ETH, one isolate with discordant results was to be false resistance, giving a sensitivity of 75%. This strain harbored an S266R of *ethA* mutation related to ETH resistance (Table 4). Based on the suggested concentration for ETH, MGIT960 test results compare poorly with those of the L-J method, at 86.2% concordance. Although the 86.2% agreement was found, this population with resistant is small. This discordance is not a novel observation, although its extent is larger than that observed in previous studies [12, 28]. Acute ETH DST has always been difficult to obtain because the drug is thermolabile [29]. ETH is an important drug for the treatment of MDR-TB and required for further DST testing and studies. 

For PAS, an MIC of 4.0 *μ*g/mL was reported [19]. PAS was tested among 0.5~16.0 *μ*g/mL in this study (Table 2).

PAS, when applying the 2.0 *μ*g/mL for the MGIT method, was 91% concordant (94.1% sensitivity, 87.5% specificity). One discrepant strain from new cases patients had low-level resistance (at 0.5 *μ*g/mL) but susceptible at >1.0 *μ*g/mL. 

The agreement for ETH and PAS was low, since we were not able to decide the concentration at which 100% of the resistant strains grew in the presence of the drug and 100% of the susceptible strains were inhibited. Larger studies are needed to define the further susceptibility test. 

In summary, MGIT960 and L-J proportion methods for second-line DST for *M. tuberculosis* were established for six drugs. The critical concentration for moxifloxacin was determined 0.5 *μ*g/mL, for levofloxacin 1.0 *μ*g/mL, for kanamycin 2.5 *μ*g/mL, for capreomycin 2.5 *μ*g/mL, for ethionamide 5.0 *μ*g/mL, and *ρ*-aminosalicylic acid 2.0 *μ*g/mL using MGIT 960. These critical concentrations were reliable for testing 2nd line drug resistance. The two drugs whose ETH and PAS were low with agreement are required for further DST testing and studies.

## Figures and Tables

**Table 1:** Clinical *M. tuberculosis* strains used in the study.

Test drugs	Number of strains	
	Susceptible	Resistant^a^
MXF		
New cases	37	4
Previously treated cases	3	15
LEV		
New cases	40	4
Previously treated cases	0	15
KM		
New cases	40	6
Previously treated cases	0	10
CPM		
New cases	39	5
Previously treated cases	1	6
ETH		
New cases	39	2
Previously treated cases	0	6
PAS		
New cases	40	6
Previously treated cases	0	11

^a^Treatment period of drug in resistant strains was >6 months.

**Table 2:** Drug concentrations tested in MGIT 960 and L-J proportion method.

Drug	Concentration evaluated (*μ*g/mL)	
	MGIT960	L-J
Moxifloxacin	0.25, 0.5, 1.0, 2.0, 4.0	2.0
Levofloxacin	0.25, 0.5, 1.0, 2.0, 4.0, 8.0	2.0
Kanamycin	0.625, 1.25, 2.5, 5.0, 6.0, 10.0	40.0
Capreomycin	0.625, 1.25, 2.5, 5.0	40.0
Ethionamide	0.625, 1.25, 2.5, 5.0	40.0
*ρ*-aminosalicylic acid	0.5, 1.0, 2.0, 4.0, 8.0, 16.0	1.0

**Table 3:** Drug susceptibility testing results of each drug determined by use of the MGIT 960 system to be compared with L-J.

Drug	Conc. evaluated (*μ*g/mL)	No. of strains with indicated results by L-J/MGIT 960				Sensitivity (%)	Specificity (%)
		*R*/*R*	*S*/*S*	*R*/*S*	*S*/*R*		
MXF	0.5	19	36	0	4	100	90.0
LEV	1.0	18	37	1	3	94.7	92.5
KM	2.5	16	38	0	2	100	95.0
CPM	2.5	10	37	1	3	90.9	90.3
ETH	5.0	6	38	2	1	75.0	97.4
PAS	2.0	16	35	1	5	94.1	87.5

**Table 4:** Drug susceptibility and genotypic characterization of isolates show discrepant results between L-J and Bactec MGIT 960.

Discordant isolates	Susceptibility at the following concentration (*μ*g/mL)				KIT number	Genotypic characterization
MXF	0.25	0.5	1.0			
*S*/*R*	*R*	*R*	*S*		1176, 1503, 1574	*gyrA*, wt; *gyrB*, wt
	*R*	*R*	*R*		1575^a^	

LEV	0.5	1.0	2.0			
*R*/*S*	*R*	*S*	*S*		2933^b^	*gyrA*, wt; *gyrB*, wt
*S*/*R*	*R*	*R*	*S*		1147, 1155, 4900	

KM	1.25	2.5	5.0			
*S*/*R*	*R*	*R*	*R*		1562, 1565	*rrs*, wt

CPM	0.625	1.25	2.5	5.0		
*R*/*S*	*R*	*S*	*S*	*S*	4831	*rrs*, wt; *tlyA*, wt
*S*/*R*	*R*	*R*	*R*	*S*	1168, 1182, 2809	

ETH	1.25	2.5	5.0			
*R*/*S*	*R*	*S*	*S*		5189	*ethA*, *ethR*, *inhA*, wt
	*R*	*R*	*S*		5298^c^	*ethA*, S266R; *ethR*, *inhA*, wt
*S*/*R*	*R*	*R*	*R*		2934	*ethA, ethR, inhA, wt *

PAS	0.5	1.0	2.0	4.0		
*R*/*S*	*R*	*S*	*S*	*S*	4891	
*S*/*R*	*R*	*R*	*R*	*S*	1201, 1212, 1214, 1573	Not done
	R	R	R	R	4537	

^a^Determined as a false-resistant result.

^b^Treatment period of drug in this patient was 17 months.

^c^Determined as a false-susceptible result.
